# Anterior Foregut Microbiota of the Glassy-Winged Sharpshooter Explored Using Deep 16S rRNA Gene Sequencing from Individual Insects

**DOI:** 10.1371/journal.pone.0106215

**Published:** 2014-09-03

**Authors:** Elizabeth E. Rogers, Elaine A. Backus

**Affiliations:** United States Department of Agriculture, Agricultural Research Service, San Joaquin Valley Agricultural Sciences Center, Parlier, California, United States of America; International Atomic Energy Agency, Austria

## Abstract

The glassy-winged sharpshooter (GWSS) is an invasive insect species that transmits *Xylella fastidiosa*, the bacterium causing Pierce's disease of grapevine and other leaf scorch diseases. *X. fastidiosa* has been shown to colonize the anterior foregut (cibarium and precibarium) of sharpshooters, where it may interact with other naturally-occurring bacterial species. To evaluate such interactions, a comprehensive list of bacterial species associated with the sharpshooter cibarium and precibarium is needed. Here, a survey of microbiota associated with the GWSS anterior foregut was conducted. Ninety-six individual GWSS, 24 from each of 4 locations (Bakersfield, CA; Ojai, CA; Quincy, FL; and a laboratory colony), were characterized for bacteria in dissected sharpshooter cibaria and precibaria by amplification and sequencing of a portion of the 16S rRNA gene using Illumina MiSeq technology. An average of approximately 150,000 sequence reads were obtained per insect. The most common genus detected was *Wolbachia*; sequencing of the *Wolbachia ftsZ* gene placed this strain in supergroup B, one of two *Wolbachia* supergroups most commonly associated with arthropods. *X. fastidiosa* was detected in all 96 individuals examined. By multilocus sequence typing, both *X. fastidiosa* subspecies *fastidiosa* and subspecies *sandyi* were present in GWSS from California and the colony; only subspecies *fastidiosa* was detected in GWSS from Florida. In addition to *Wolbachia* and *X. fastidiosa*, 23 other bacterial genera were detected at or above an average incidence of 0.1%; these included plant-associated microbes (*Methylobacterium*, *Sphingomonas*, *Agrobacterium*, and *Ralstonia*) and soil- or water-associated microbes (*Anoxybacillus*, *Novosphingobium*, *Caulobacter*, and *Luteimonas*). Sequences belonging to species of the family Enterobacteriaceae also were detected but it was not possible to assign these to individual genera. Many of these species likely interact with *X. fastidiosa* in the cibarium and precibarium.

## Introduction

*Homalodisca vitripennis* (Germar) (Hemiptera: Cicadellidae: Cicadellinae) or glassy-winged sharpshooter (GWSS) is a leafhopper that exclusively ingests plant xylem fluid from a wide range of host plants [1]. GWSS is considered an important pest in commercial agriculture because it, along with other sharpshooter species, transmits the phytopathogenic bacterium *Xylella fastidiosa* [2]. *X. fastidiosa* is the causative agent of a variety of scorch diseases including Pierce's disease (PD) of grapevine, almond leaf scorch disease (ALSD), and citrus variegated chlorosis (CVC). *X. fastidiosa* is unique as the only known arthropod-transmitted plant pathogen that is both propagative and non-circulative, multiplying solely in the insect's anterior foregut without becoming circulative in the insect's hemolymph [3].

Morphologically, the functional anterior foregut of a sharpshooter consists of: 1) the precibarium, a narrow channel that conveys fluid from the stylets (piercing-sucking mouth parts) into 2) the cibarium, the sucking pump, from which the fluid is swallowed into the rest of the gut. Studies of the cibarium and precibarium, using both light and electron microscopy, have shown patch-like colonies of bacteria interspersed with naked cuticle. The earliest microscopy study used both light and scanning electron microscopy (SEM) to show rod-shaped bacteria embedded in a gum-like matrix in inoculative, i.e. competent to inoculate pathogen, but not in non-inoculative, *Graphocephala atropunctata* (Signoret) (blue-green sharpshooter; BGSS) [4]; these bacteria were assumed to be the PD bacterium (now known as *X. fastidiosa*). Later SEM studies found similar bacteria in *Oncometopia nigricans* (Walker) and *H. vitripennis* individuals that had fed on plants colonized by *X. fastidiosa* [5]; sharpshooters fed on healthy plants were reported to be bacteria-free. A more recent SEM study of *G. atropunctata* again demonstrated that the presence of bacteria in the cibarium and precibarium was highly correlated with infectivity [6]. Three confocal light microscopy studies introducing and visualizing fluorescent protein-expressing bacteria (either *X. fastidiosa* or an *Alcaligenes* sp.) have shown single bacterial cells and small clumps or colonies of cells in the anterior foregut of inoculative sharpshooters [3], [7], [8].

Several small-scale studies of bacterial 16S sequences associated with sharpshooters have previously been published. In 2003, Moran *et al.* [9] dissected bacteriomes from several species of Cicadellinae and detected a novel symbiont they named “*Candidatus* Baumannia cicadellinicola.” Subsequent work from the same group identified another novel symbiont, “*Candidatus* Sulcia muelleri,” associated with various sharpshooters and other related insects [10], [11]. *B. cicadellinicola*, *W. pipientis*, as well as bacteria closely related to *Pseudomonas*, *Stenotrophomonas* and *Acintobacter* were identified in GWSS hemolymph in an independent study [12]. Lacava *et al.*, 2007 [13] used a combination of denaturing gradient gel electrophoresis, culturing, and 16S sequencing to characterize bacteria associated with GWSS heads. In a larger 16S sequencing study, Hail et al., 2011 [14] detected predominantly *Pectobacterium* in hemolymph and *Cardiobacterium* associated with the alimentary canal and whole insect. In contrast to previous 16S sequencing studies, *Wolbachia* was not detected in the hemolymph, only in whole insects [14]. These previous studies have been limited in the number of sequences examined and have not compared insects from different locations; therefore, details about the extent of microbiome variation among GWSS individuals and geographic locations are unknown.

This study is the first to use next-generation sequencing (NGS) techniques to exhaustively catalog bacteria associated with the GWSS cibarium and precibarium. A short, diagnostic region of the bacterial 16S RNA gene was amplified by PCR from 96 individual insects (24 each from 4 different geographic locations). Illumina sequencing technology was used to pool PCR products and obtain at least 50,000 individual reads per insect. To more precisely identify a few common bacterial genera (*Wolbachia*, *Xylella*, *Anoxybacillus*, *Caulobacter*, and *Novosphingobium*), additional Sanger sequencing is presented. Cataloging foregut-associated bacteria is a necessary first step toward examining interactions among bacterial species in GWSS foregut and addressing the hypothesis that other bacterial species can affect the ability of a sharpshooter to acquire, retain, and/or inoculate *X. fastidiosa*.

## Results

### 16S rRNA Sequencing

In total, more than 19 million paired-end reads were recovered using Illumina-based sequencing (Table 1). Approximately 92% of these reads passed Illumina's stringent quality control (QC) measures and of those, just over 84% could be classified as bacterial 16S amplicons (including 16S sequences from chloroplasts). Demultiplexing, where the dual-end indices are used to assign each read to an individual insect, was performed using Illumina's MiSeq Reporter software, giving between about 50,000 and one million high quality reads per insect sample (Table 1).

**Table 1:** Summary of Sequencing Results.

All Sequences:	total	average	low	high
# raw reads retrieved:	19,473,000			
# reads passing quality control:	17,882,483	186,276	57,736	1,045,276
unclassified (not 16S):	2,838,274	29,565	5,343	105,435
classified (bacterial 16S):	15,044,209	156,711	49,833	943,506

The number of sequencing reads (total, average per insect, lowest value, and highest value) are presented.

### Assignment of Bacterial Classifications

**Figure 1:**
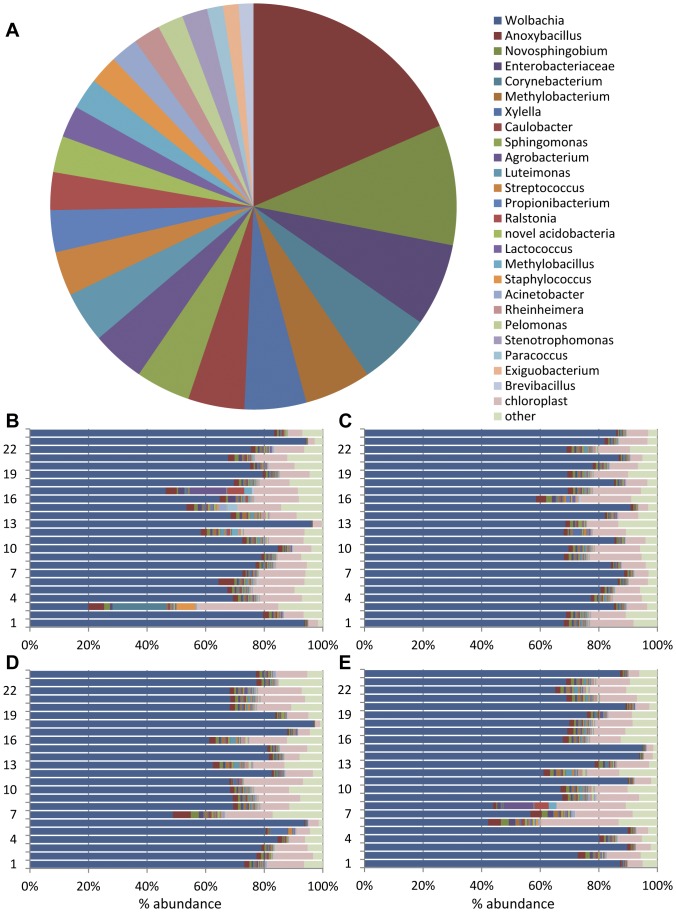
Genera most commonly associated with *H. vitripennis* cibarium and precibarium. A) Average abundance of non-*Wolbachia* genera over all 96 insects; B–E) Abundance of all classifications from B) Bakersfield individuals; C) Ojai individuals; D) Florida individuals; E) colony individuals. Color legend in panel A applies to all panels (A–E); order of classifications in the legend is the same as the order around the pie graph in A (starting with the dark red *Anoxybacillus* in the upper right) and in the bar graphs B–E (starting with the dark blue *Wolbachia* on the left).

MiSeq Reporter automatically assigns taxonomic classifications to reads by comparing sequences to an Illumina-curated version of the Greengenes 16S rRNA gene database. In addition, FastQ files were exported from MiSeq Reporter and Genomics Workbench was used to align paired ends, remove primer sequence, and group reads into operational taxonomic units (OTUs) of at least 97% identity. Taxonomic classifications were assigned to OTUs using the BLAST function within Genomics Workbench. These two methods gave very similar results; the number of reads assigned to either *Wolbachia* or to *X. fastidiosa* varied by less than 2% for all 96 insects. To compare taxon and OTU prevalence between insects, percent abundance was calculated based on total number of classified reads. In addition, annotation of each genus was manually checked by BLAST (www.ncbi.nlm.nih.gov) against both the non-redundant and reference RNA sequence databases. Taxonomic classifications were identical except for the OTU listed as “novel acidobacteria” in Table 2. MiSeq Reporter assigned that group to the genus *Candidatus* Solibacter. However, manual BLAST searches revealed only 93% identity between the OTU and published *Solibacter usitatus* Ellin6076 16S sequence. Since this is below the 97% identity threshold for OTU formation, this group was relabeled “novel acidobacteria.” Its consensus 16S sequence is 99% identical to several uncultured bacterium clones from two separate unpublished studies, one of a constructed wetland in France and the other from Tibetan permafrost (i.e. accession numbers KC432438 and JQ684311). Many genera from the family Enterobacteriaceae have identical V6 regions of the 16S rRNA gene; therefore it was not possible to identify this OTU below the family level. The MiSeq Reporter results are presented in Table 2 as average % abundance over all 96 samples. The category “other/unclassified” represents taxa present at less than 0.1% or reads that were not sufficiently similar to known 16S genes to be mapped to a genus. Because Wolbachia is likely not present in the cibarium and precibarium, it, along with chloroplast and other/unclassified, were omitted from Figure 1A in order to better demonstrate the diversity and abundance of identified bacterial genera potentially present in precibaria and cibaria; all classifications are included in Figure 1B–E to convey inter-insect variability.

**Table 2:** Bacterial Genera Abundance.

Classifications:	average (%)	SD (%)
*Wolbachia*	74.55	12.98
*Anoxybacillus*	1.54	1.07
*Novosphingobium*	0.80	0.49
Enterobacteriaceae	0.55	0.44
*Corynebacterium*	0.49	1.85
*Methylobacterium*	0.44	0.32
*Xylella*	0.41	0.65
*Caulobacter*	0.37	0.20
*Sphingomonas*	0.36	0.23
*Agrobacterium*	0.35	1.63
*Luteimonas*	0.34	0.50
*Streptococcus*	0.29	0.20
*Propionibacterium*	0.28	0.20
*Ralstonia*	0.25	0.75
novel acidobacteria	0.24	0.14
*Lactococcus*	0.21	0.17
*Methylobacillus*	0.21	0.39
*Staphylococcus*	0.19	0.66
*Acinetobacter*	0.18	0.27
*Rheinheimera*	0.18	0.27
*Pelomonas*	0.17	0.23
*Stenotrophomonas*	0.17	0.15
*Paracoccus*	0.11	0.34
*Exiguobacterium*	0.10	0.08
*Brevibacillus*	0.10	0.09
chloroplast	10.15	5.23
other/unclassified	6.99	3.65

All bacterial genera present at or above 0.1% of the total are listed. Data are from MiSeq Reporter; classifications were verified by BLAST.

### α- and β-Diversity

**Figure 2:**
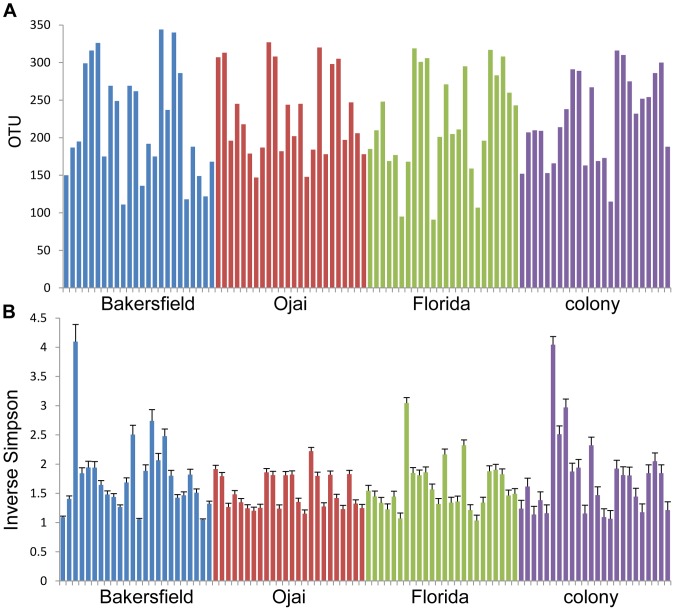
α Diversity Measures. Total number of OTU observed per insect (A) and inverse Simpson index (B) for each of the 96 insects as calculated using mothur. Error bars in B are 95% confidence levels. Bars are color-coded: blue (Bakersfield), red (Ojai), green (Florida), and purple (colony).

The total number of OTUs observed in each of the 96 insects is shown in Figure 2A; Figure 2B shows the inverse Simpson index, another measure of α-diversity or richness of each individual microbiome. Examining β-diversity, AMOVA (analysis of molecular variance) showed no significant differences among the bacterial profiles from each of the four locations (Fs = 1.14, df = 95, p-value = 0.337). To examine potential geographical differences, the California 72 insects (24 from Bakersfield, 24 from Ojai, and 24 colony) were grouped together and compared with the 24 Florida insects also using AMOVA. Again, no significant differences were observed (Fs = 0.438, df = 95, p-value = 0.579).

### *Wolbachia ftsZ* gene sequence

**Figure 3:**
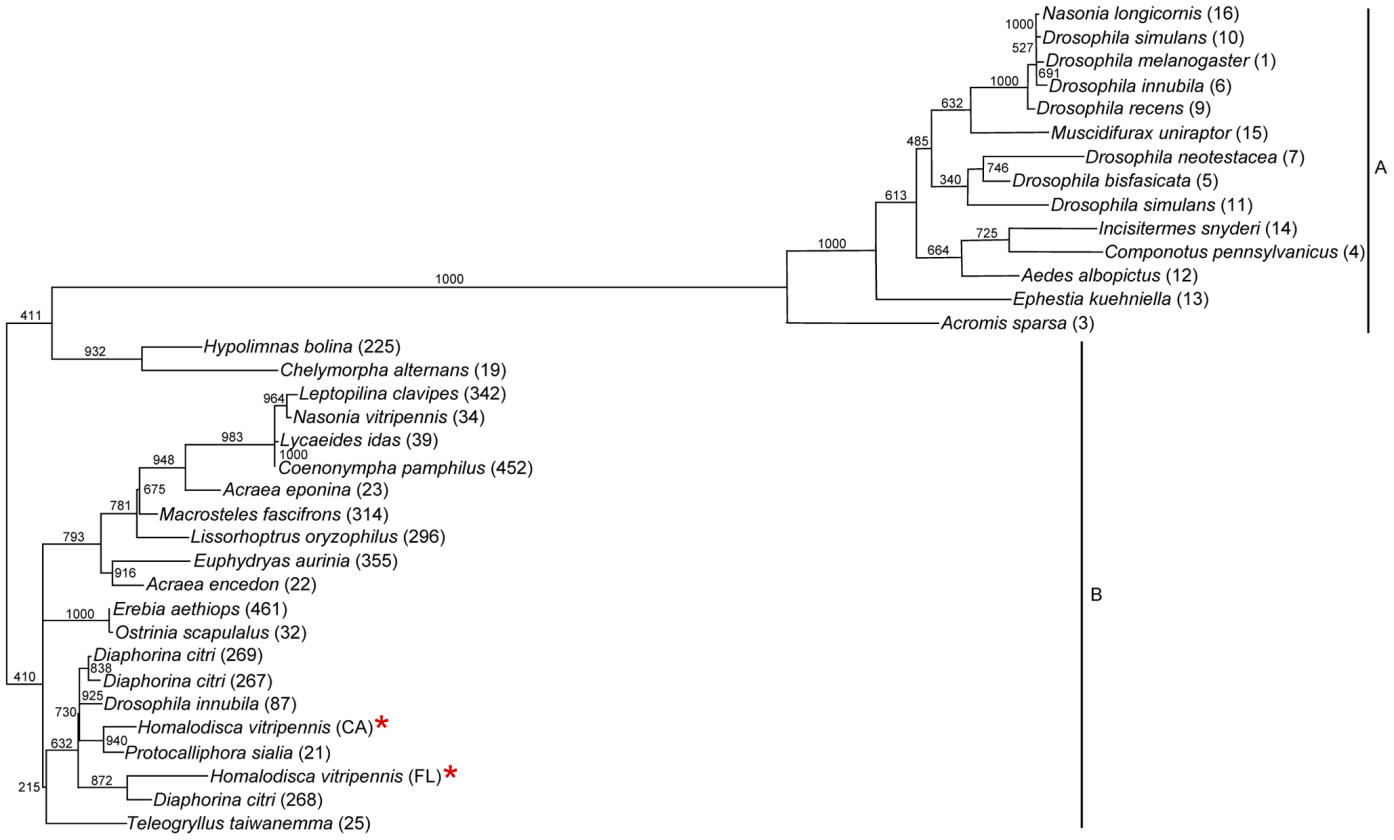
Phylogeny of Wolbachia MLST genes from various insects. A maximum likelihood tree (1000 bootstrap replicates) is based on multiple alignments of concatenated DNA sequences encoding the *coxA*, *fbpB*, *ftsZ*, *gatB*, *hcpA*, and *wsp* genes. Bootstrap values are shown for all nodes; red asterisks indicate sequences from this study. Sequence types from pubmlst.org/wolbachia are given in parentheses. Lines to the right indicate Wolbachia supergroups A and B.

The 16S rRNA gene shows little to no divergence in many strains of *Wolbachia* isolated from disparate insect hosts and geographic locations. MLST (multilocus sequence typing) analysis been used to further characterize *Wolbachia* strains [15]. Sequences from the three California insect sources (Bakersfield, Ojai, and the colony, which was originally collected in Bakersfield) were identical and are grouped together in Table 3. However, there are quite a few polymorphisms between the California insects and the Florida ones, especially in the *coxA* and *wsp* loci. A larger, overlapping region of the *ftsZ* gene was also cloned by PCR and sequenced [16]. There were no differences in this longer *ftsZ* sequence among the four geographic locations. As shown in Figure 3, the MLST sequences determined here place the *Wolbachia* from *H. vitripennis* in supergroup B of *Wolbachia pipientis* [16], [17]. *W. pipientis* supergroups A and B are most commonly associated with arthropod species.

**Table 3:** *Wolbachia* MLST Results.

loci	California	Florida	bp / aa differences between CA and FL
*gatB*	9	227*	3
*coxA*	212*	221*	14
*hcpA*	88	251*	1
*ftsZ*	195*	195*	0
*fbpA*	27	403*	2
*wsp*	378	675*	59^a^
HVR1	150	233*	4
HVR2	165	266*	10^b^
HVR3	24	8	7
HVR4	3	7	10

a 59 differences include 56 substitutions and 3 indels (3 bp each).

b 10 differences include 8 substitutions and 2 indels (1 aa each).

Five individual cloned PCR products were sequenced for each locus from each insect population. Sequences from all three California populations (Bakersfield, Ojai, and colony) were identical and have been grouped together. Allele numbers are from http://pubmlst.org/wolbachia/ and novel alleles are marked (*). Differences are base pairs for genes (*gatB*, *coxA*, *hcpA*, *ftsZ*, *fbpA*, and *wsp*) and amino acids for peptides (HVR1, 2, 3 and 4).

### *X. fastidiosa* MLST Analysis and Quantitative PCR

**Figure 4:**
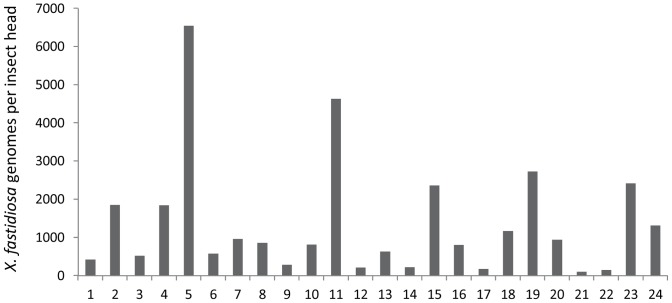
*X. fastidiosa* genomes per GWSS head. Quantitative RT-PCR was used to measure the number of *X. fastidiosa* genomes associated with 24 different dissected GWSS heads from the colony.

MLST has also been used to characterize *X. fastidiosa* strains and assign subspecies [18], [19]. Due to limited amounts of starting DNA, only 4 of the typical 8 loci were sequenced (*pilU*, *petC*, *leuA*, and *gltT*). As shown in Table 4, sequences corresponding to both the *fastidiosa* and *sandyi* subspecies were detected in GWSS from both California locations and from the colony; however, only the *fastidiosa* subspecies was detected in Florida GWSS. It is noteworthy that subspecies *sandyi* has not been found in Florida. *X. fastidiosa* levels were quantitated using real time PCR on DNA extracted from 24 GWSS from the colony. As shown in Figure 4, *X. fastidiosa* genomes per dissected insect head ranged from 100 to 6500 (average 790). Due to the limited amount of DNA in a dissected GWSS head, these are different insects than those used for 16S rRNA sequencing.

**Table 4:** *Xylella fastidiosa* MLST Results.

Loci	Bakersfield	Ojai	Florida	Colony
*pilU*	*fastidiosa* (1) *sandyi* (4)	*fastidiosa* (5)	*fastidiosa* (5)	*sandyi* (5)
*petC*	*fastidiosa* (3) *sandyi* (2)	*fastidiosa* (2) *sandyi* (3)	*fastidiosa* (5)	*fastidiosa* (5)
*leuA*	*fastidiosa* (3) *sandyi* (2)	*sandyi* (5)	n.a.*	*fastidiosa* (1) *sandyi* (4)
*gltT*	*fastidiosa* (3) *sandyi* (2)	*fastidiosa* (1) *sandyi* (4)	*fastidiosa* (5)	n.a.*

* n.a. = no amplification.

Five individual cloned PCR products were sequenced for each locus from each insect population. Numbers in parentheses are the number of times sequences of each subspecies were detected. Due to limited amounts of DNA, *leuA* in the Florida population and *gltT* from the colony were not amplified.

### Additional Sequencing of the 16S rRNA genes from *Anoxybacillus*, *Caulobacter*, and *Novosphingobium*

To further characterize the species present, additional sequencing of the 16S rRNA gene was performed for these three genera. Sequences were compared to the GENBANK non-redundant DNA database by BLAST (www.ncbi.nlm.nih.gov). For *Anoxybacillus*, the longer 16S sequences did not allow assignment to the species level. Sequences obtained here, which did not vary among the four geographic locations, are identical to 27 sequences; 10 of these are annotated as *Anoxybacillus* (*A. flavithermus*, *A. kamchatkensis*, or unspecified *Anoxybacillus* spp.). Many Anoxybacilli are found in hot springs but some are insect-associated (e.g. JQ894876 from spiraling whitefly or KC170396 from the aphid *Cinara tujafilina*) or plant-associated (e.g. KC478840 from Indian mulberry *Morinda citrifolia*).

The *Caulobacter* sequence obtained here is 100% identical to 49 sequences in GENBANK, all uncultured and annotated as *Caulobacter* spp. or *C. henricii*. These sequences are from such diverse habitats as endosphere of hybrid poplar, coral and coral reef water, sea floor, soil, the South American moth *Rothschildia lebeau* larva, citrus trees, rice seeds, and rice paddy soil. The *Novosphingobium* sequence obtained here is 100% identical to 6 sequences in GENBANK: one uncultured from Artic seawater; and 5 cultured bacterial strains from rice roots and paddy soil or sugarcane and associated soils. By comparison to the NCBI reference RNA database, the sequence obtained here is 99% identical to *Novosphingobium resinovorum* strain NCIMB 8767 originally isolated from soil.

## Discussion

Bacteria have been found associated with the guts of many insect species [20]. Therefore it is not surprising that so many disparate bacterial genera were found associated with GWSS cibarium and precibarium. *Wolbachia* is a common insect pathogen and has been detected in GWSS and other sharpshooters previously [10], [11], [13], [14]. Because *Wolbachia* is an endosymbiont [17], the *Wolbachia* detected here most probably comes from cellular contents or hemolymph liberated during the dissection process.

Many of the non-*Wolbachia* genera found in this study have been detected previously in association with plants or soil: *Novosphingobium*, *Sphingomonas*, *Methylobacterium*, *Methylobacillus*, *Agrobacterium*, *Ralstonia*, and *Lutemonas*. The casparian strip is an impermeable barrier in a root that separates the bulk soil solution and vascular fluids. However, it is possible for solutes and bacterial cells present in soil to passively enter the root xylem stream through lateral root junctions, wounds or root tips, which lack a casparian strip [21]. Therefore, it is not surprising to find soil bacteria present in xylem or in an exclusively xylem-ingesting insect.

The presence of chloroplast DNA may indicate that cellular contents of non-xylem cells are taken up into GWSS precibarium and cibarium during plant feeding. It has been hypothesized [22] that sharpshooters take up fluid into the precibarium from non-xylem cells encountered along the stylet pathway prior to reaching a xylem vessel. Perhaps uptake of non-xylem cell contents is performed to taste chemical constituents using gustatory sensilla lining the precibarium [23], and may aid in orienting the stylets towards xylem. Chloroplast DNA also likely enters the xylem stream through wounds and as part of the xylem maturation process. It is possible that chloroplasts brought into the precibarium lodged in bacterial biofilm colonizing the cuticle and thus were retained.

Several of the bacterial genera detected here have been previously reported to be sharpshooter-associated: *Exiguobacterium*, *Stenotrophomonas*, *Pelomonas*, *Acinetobacter*, *Ralstonia*, and *Methylobacterium* [12]–[14]. Sequences consistent with the previously reported GWSS endosymbionts *Baumannia cicadellinicola* and *Sulcia muelleri* were detected here at levels below 0.1% average abundance [9], [10]. It is likely these endosymbionts originated in hemolymph liberated during dissection, but at very low levels because they normally reside intracellularly in abdominal bacteriomes [9]. The Enterobacteriaceae includes the human pathogens *Escherichia*, *Salmonella*, and *Shigella*. Therefore it is possible that sequences from this family represent contaminants introduced during insect handling, dissection, or DNA processing. Similarly, *Proprionibacterium* has been most commonly found associated with human skin and also may be a contaminant.

It is not possible to directly calculate numbers of bacterial cells per insect head from 16S sequencing data. However, there is good correlation between percent abundance and copy number as determined by quantitative PCR (qPCR) [24]. There was not sufficient DNA to perform qPCR on GWSS sampled here; however, qPCR performed on other GWSS from the laboratory colony typically detected approximately 800 *X. fastidiosa* per insect (Figure 4).

Several microscopy studies have described uniform “shag carpets” of bacteria lining the cibaria and precibaria in inoculative sharpshooters and have hypothesized these are pure cultures of *X. fastidiosa*. Data presented here suggests there may be other bacteria in these locations, including other gram-negative rods that would be very similar in appearance to *X. fastidiosa*. *Novosphingobium*, *Sphingomonas*, *Caulobacter*, *Methylobacterium*, *Methylobacillus*, *Agrobacterium*, *Ralstonia*, and *Lutemonas* are all gram-negative, rod-shaped bacteria. It cannot be stated with certainty that any non-*X. fastidiosa* bacteria detected here attached to or actively colonized the precibarial and cibarial cuticle. Consequently, microscopy studies of appropriately labeled bacteria would be needed to provide definitive proof of multiple bacterial species binding to the cuticle of sharpshooter cibarium and/or pre-cibarium.

## Materials and Methods

### Insect Collection and Dissection

*H. vitripennis* were field-collected from: 1) oleander (*Nerium oleander* (L.)) and photinia (*Photinia* spp.) plants in Bakersfield, CA on June 21, 2012 (Bakersfield), 2) citrus in Ojai, CA on July 24, 2012 (Ojai), and 3) wild sunflower (*Helianthus annuus* (L.)) in Quincy, FL on August 31, 2012 (Florida). Insects also were taken from 4) a *H. vitripennis* colony maintained in Parlier, CA on October 23, 2012 (colony). Colony insects were originally collected on oleander and photinia in Bakersfield, CA in 2003–2005, and maintained since then under artificial conditions (24–30°C, 16∶8 L∶D) without diapause, on a mixture of cowpea (*Vigna unguiculata* (L.)), sorghum (*Sorghum bicolor* (L.)), sweet basil (*Ocimum basilicum* (L.)), and sunflower plants in the same cage. *H. vitripennis* is not an endangered or protected species; permission to collect *H. vitripennis* was given by land owners in all locations.

Field-collected insects were frozen whole while colony insects were decapitated and only the heads were frozen; insect tissue was stored at −20°C until dissection. Twenty-four individuals from each geographic location were rapidly dissected on a cold plate to reduce thawing, using a Leica MZ16 stereoscan microscope, until only the precibarium and cibarium of the anterior foregut remained. Precautions were taken to maintain sterile technique and to avoid contamination of the sample. Dissected precibarium and cibarium from each individual insect was refrozen at −20°C until DNA isolation was performed according to a previously described protocol [25].

### Construction of Libraries and DNA Sequencing

PCR primers were designed to amplify approximately 200 bp spanning V6 (the 6^th^ variable region) of the 16S ribosomal RNA gene. In order to provide the nucleotide diversity needed by Illumina sequencing platforms, 6 random nucleotides were added to the 5′ end of each primer. Additional diversity was created using pools for 6 forward and 6 reverse primers where the start of each primer was shifted by one bp (Table 5). Ninety-six individual PCR reactions were performed using the Expand High Fidelity PCR System (Roche Applied Science) in an S1000 Thermal Cycler (BioRad) according to manufacturers' directions. PCR products were purified using the QIAquick PCR Purification Kit (Qiagen). Libraries were constructed by ligating sequencing adapters and indices onto purified PCR products using the TruSeq DNA HT Sample Preparation Kit (Illumina). This kit adds one of 12 indices (unique 8 bp sequence tags) to the forward end and one of 8 different indices to the reverse end for 96 unique index combinations. Libraries were quantitated using a KAPA Library Quantification Kit (Kapa Biosystems) in a StepOnePlus Real Time PCR System (Applied Biosystems). Equimolar amounts of each of the 96 libraries were pooled and submitted for sequencing on a MiSeq Personal Sequencer using a 150-bp paired-end protocol at the University of Georgia Genomics Facility.

**Table 5:** Primers used to amplify 16S DNA.

Forward primers	Reverse primers
NNNNNNAAACTCAAAGGAATTGACGG	NNNNNNGGGTTGCGCTCGTTGCGG
NNNNNNAACTCAAAGGAATTGACGGG	NNNNNNGGTTGCGCTCGTTGCGGG
NNNNNNACTCAAAGGAATTGACGGGG	NNNNNNGTTGCGCTCGTTGCGGGA
NNNNNNCTCAAAGGAATTGACGGGGR	NNNNNNTTGCGCTCGTTGCGGGAC
NNNNNNTCAAAGGAATTGACGGGGRC	NNNNNNTGCGCTCGTTGCGGGACT
NNNNNNCAAAGGAATTGACGGGGRCC	NNNNNNGCGCTCGTTGCGGGACTT

Equimolar amounts of all 6 forward primers and all 6 reverse primers were pooled and used in PCR. Forward primers span nt 926–950; reverse primers span nt 1107–1129 (numbering relative to *E. coli* 16S DNA).

### DNA Sequence Analysis

Sequence analysis, including demultiplexing and removal of indices, was performed using the bacterial metagenomics workflow in the MiSeq Reporter software (Illumina). FastQ sequence files (after demultiplexing and index removal) were also exported from MiSeq Reporter for further analysis using CLC Genomics Workbench (CLC Bio). The two analysis methods gave very similar results; discrepancies are noted in the Results. Inverse Simpson indices and AMOVAs (analysis of molecular variance) were calculated using the mothur package: www.mothur.org [26], [27]. To construct the phylogenetic tree, concatenated MLST sequences were downloaded from http://pubmlst.org/wolbachia and aligned with sequences from this study using ClustalW at Biology Workbench 3.2 (http://seqtool.sdsc.edu/CGI/BW.cgi) [28]. Maximum likelihood tree was drawn using T-REX (http://www.trex.uqam.ca) [29], [30]. The fastQ sequence files have been deposited at MG-RAST (http://metagenomics.anl.gov/metagenomics.cgi?page=Home) with ID # 4522303–4522398. A summary of the raw data is presented in Table S1.

### Wolbachia *ftsZ* and MLST sequencing

All 24 original DNA samples from each location were pooled and PCR was performed using the previously described *fts*ZUNIF and *fts*ZUNIR primers [16] and MLST primers [15]. PCR products were cloned using a TOPO TA Cloning Kit with pCR2.1 (Life Technologies). Five individual clones from each location were sequenced using the M13 forward and reverse primers on a 3130×l Genetic Analyzer (Applied Biosystems). Sanger sequence data were analyzed using Sequencher 5.1 (Gene Codes Corporation). Final *ftsZ* sequence was deposited in GenBank (accession number KF636751) and final MLST sequences were uploaded to http://pubmlst.org/wolbachia/.

### Additional sequencing of selected 16S genes

The Illumina sequences were used to design specific primers to the V6 region of the 16S gene for three common genera: *Anoxybacillus* (AB-F CCCCTGACAACCCGAGAGATCGGGCG), *Caulobacter* (CA-F GCCCGGACCGCCACAGAGATGTGGCTT), and *Novosphingobium* (NS-F CCCGCGCTACACAGAGAGATTTGTGG). These primers were used along with a 1544R universal 16S DNA primer (AAGGAGGTGATCCAGCC) to amplify approximately 600 bp spanning variable regions 7, 8, and 9 of selected 16S DNAs. As with the Wolbachia MLST, PCR products were cloned into pCR2.1 and five individual clones of each gene from each location were sequenced.

### *X. fastidiosa* MLST sequencing and quantitative PCR

Previously reported primers were used to amplify portions of the *pilU*, *petC*, *leuA*, and *gltT* genes [19] from pooled DNA from each location. As with the Wolbachia MLST, PCR products were cloned into pCR2.1 and five individual clones of each gene from each location were sequenced. Subspecies were assigned using the *Xylella fastidiosa* MLST Databases at pubmlst.org/xfastidiosa/. Quantitative real-time PCR was performed on a StepOnePlus Real Time PCR System (Applied Biosystems) as previously described [25].

## Supporting Information

Table S1 **OTU Table.** Number of times each genera was detected in each of the 96 insects.
(XLSX)
